# Unique Peptides of Cathelicidin-1 in the Early Detection of Mastitis—In Silico Analysis

**DOI:** 10.3390/ijms241210160

**Published:** 2023-06-15

**Authors:** Maria V. Bourganou, Evangelos Kontopodis, George Th. Tsangaris, Vasileios Pierros, Natalia G. C. Vasileiou, Vasia S. Mavrogianni, George C. Fthenakis, Angeliki I. Katsafadou

**Affiliations:** 1 Faculty of Public and One Health, University of Thessaly, 43100 Karditsa, Greece; 2 Proteomics Research Unit, Biomedical Research Foundation of the Academy of Athens, 11527 Athens, Greece; 3 Faculty of Animal Science, University of Thessaly, 41110 Larissa, Greece; 4 Veterinary Faculty, University of Thessaly, 43100 Karditsa, Greecegcf@vet.uth.gr (G.C.F.)

**Keywords:** biomarker, cathelicidin-1, diagnosis, mastitis, sheep, unique peptide

## Abstract

Based on the results of previously performed clinical studies, cathelicidin-1 has been proposed as a potential biomarker for the early diagnosis of mastitis in ewes. It has been hypothesized that the detection of unique peptides (defined as a peptide, irrespective of its length, that exists in only one protein of a proteome of interest) and core unique peptides (CUPs) (representing the shortest peptide that is unique) of cathelicidin-1 may potentially improve its identification and consequently the diagnosis of sheep mastitis. Peptides of sizes larger than those of the size of CUPs, which include consecutive or over-lapping CUPs, have been defined as ‘composite core unique peptides’ (CCUPs). The primary objective of the present study was the investigation of the sequence of cathelicidin-1 detected in ewes’ milk in order to identify its unique peptides and core unique peptides, which would reveal potential targets for accurate detection of the protein. An additional objective was the detection of unique sequences among the tryptic digest peptides of cathelicidin-1, which would improve accuracy of identification of the protein when performing targeted MS-based proteomics. The potential uniqueness of each peptide of cathelicidin-1 was investigated using a bioinformatics tool built on a big data algorithm. A set of CUPs was created and CCUPs were also searched. Further, the unique sequences in the tryptic digest peptides of cathelicidin-1 were also detected. Finally, the 3D structure of the protein was analyzed from predicted models of proteins. In total, 59 CUPs and four CCUPs were detected in cathelicidin-1 of sheep origin. Among tryptic digest peptides, there were six peptides that were unique in that protein. After 3D structure analysis of the protein, 35 CUPs were found on the core of cathelicidin-1 of sheep origin and among them, 29 were located on amino acids in regions of the protein with ‘very high’ or ‘confident’ estimates of confidence of the structure. Ultimately, the following six CUPs: QLNEQ, NEQS, EQSSE, QSSEP, EDPD, DPDS, are proposed as potential antigenic targets for cathelicidin-1 of sheep. Moreover, another six unique peptides were detected in tryptic digests and offer novel mass tags to facilitate the detection of cathelicidin-1 during MS-based diagnostics.

## 1. Introduction

### 1.1. Role of Cathelicidin-1 in Mastitis in Sheep

Mastitis has been reported “as an important welfare consequence in all management systems” applied in sheep farms [1,2]. The disease occurs following bacterial invasion into the mammary parenchyma and bacterial multiplication as the inflammatory reaction ensues. During subclinical mastitis, no clinical signs are evident and thus diagnosis is achieved by means of assessing the combined findings of bacteriological and cytological examination. Mammary infection proceeds rapidly and consequent damage to mammary parenchyma, with histologically evident lesions, develops within 24 h of bacterial invasion [3]. Thus, early diagnosis of subclinical mastitis is important for subsequent effective treatment and the recovery of the affected animal.

Cathelicidins refer to a major group of antimicrobial peptides that have been detected in various animal species. Cathelicidins show a direct antimicrobial activity and act against many pathogens, thus participating in the modulation of defence against the invading microorganisms. Cathelicidin proteins are part of the secondary granules of neutrophils, which are important components of the mammary defence process after bacterial invasion [4]. They are found in neutrophils and in mammary epithelial cells and increased levels of cathelicidins have been reported in the milk during the early stages of mammary invasion [5]. In such cases, cathelicidins are released extracellularly as mature peptides after the activation of leucocytes [6].

Within this context, cathelicidin-1 has been proposed as a potential biomarker for use in the early diagnosis of mastitis in ewes [5,7]. Thus, Addis et al. [8,9] proposed an ELISA assay for the rapid detection of the protein.

In a previous clinical and laboratory study performed by our group [10], we investigated the behaviour of cathelicidin-1 in the milk after experimental infection with two prominent bacterial pathogens (*Mannheimia haemolytica* and *Staphylococcus chromogenes*) as a potential early indicator for the diagnosis of mastitis in sheep. In those experiments, bacteria were inoculated into the mammary gland of ewes. Thereafter, clinical examination of animals, as well as bacteriological and cytological examinations of milk samples along with proteomics examinations of milk, were performed sequentially. Cathelicidin-1 was detected and its spot densities on two-dimensional gels were assessed and recorded (Figure S1) [10]. Associations were calculated between cathelicidin-1 spot densities and cell content in the milk (Figure S2) [10], as well as between the presence of mastitis in a mammary gland at a given time point and the detection of cathelicidin-1 in the respective milk sample (Table S1) [10]. All inoculated mammary glands developed mastitis, a fact confirmed by the consistent bacterial isolation from mammary secretion and the increased leucocyte content therein [11]. Spot densities of cathelicidin-1 in samples from inoculated glands increased 3 h post-inoculation; spot densities of cathelicidin-1 in samples from inoculated glands was higher than in samples extracted from uninoculated controls (Figure S1). There was clear evidence of correlation between cell content and cathelicidin-1 spot densities in milk samples. Also, there was a significant association between the presence of mastitis in a mammary gland and the detection of cathelicidin-1 in the respective milk sample; for this, the overall accuracy was 0.818 and was significantly greater during the first 24 h post-challenge (0.903) than after the first day (0.704).

All the above evidence supported the conclusion that the detection of cathelicidin-1 in milk was significantly associated with the presence of mastitis in ewes. In this respect, cathelicidin-1 has the advantage that it can be a non-specific biomarker, as simply a ‘positive’/‘negative’ assessment would be sufficient [10].

### 1.2. Unique Peptides and Core Unique Peptides

A ‘unique peptide’ has been defined as “a peptide, irrespective of its length, that exists only in one protein of a proteome of interest, despite the fact that this peptide may appear more than once in the same protein” [12]. In this context, Alexandridou et al. [13] introduced the concept of ‘core unique peptide’ (CUP), which represents the shortest peptide that is unique, i.e., existing only in one protein, within a set of proteins [13,14]. Peptides of sizes larger than those of the size of CUPs, which include consecutive or overlapping CUPs, have been recently termed ‘composite core unique peptides’ (CCUPs) [14].

Based on the above, the term ‘uniquome’ has been coined to include the entirety of unique peptides within a proteome [14]. Hence, in order to accurately map the unique peptides within a studied proteome, a bioinformatics tool has been developed, taking into account advanced algorithms relevant for big data analysis [14,15].

### 1.3. Hypothesis and Objectives of the Present Study

Based on the above, it has been hypothesized that the detection of unique peptides and core unique peptides of cathelicidin-1 may potentially improve its identification and consequently the diagnosis of sheep mastitis.

The primary objective of the present study was to investigate the sequence of cathelicidin-1 detected in ewes’ milk in order to identify its unique peptides and core unique peptides, which would reveal potential targets for the accurate detection of the protein. An additional objective was the detection of unique sequences among the tryptic digest peptides of cathelicidin-1, which would improve accuracy of identification of the protein during targeted MS-based proteomics.

## 2. Results

### 2.1. Reference Protein Data

In total, 460 proteins of sheep origin, 6036 proteins of cattle origin and 120 proteins of goat origin were obtained. These proteins were found by searching for the analysis of cathelicidin-1 against them. Their characteristics, in terms of total unique peptides, core unique peptides (CUPs), density of CUPs and unique coverage, are in Table S2.

**Figure 1:**
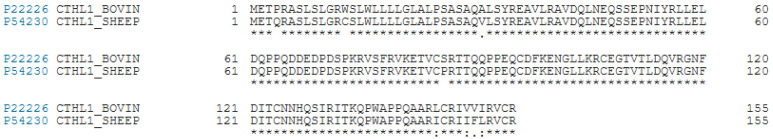
Exact amino acid and peptide sequence and alignment of cathelicidin-1 of sheep or cattle origin (marking of amino acids with (*) indicates that the residues are identical in both sequences; marking of amino acids with (.) or (:) denotes that semi-conserved substitutions or conserved substitutions, respectively, are present in the sequences; lack of marking of amino acids indicates differences between the sequences) (blue characters indicate the accession numbers of the two proteins).

The exact amino acid and peptide sequence and alignment of cathelicidin-1 of sheep and cattle origin is in Figure 1; no reviewed cathelicidin-1 of goat origin was available. In total, 147 amino acids in the two sequences were found to be identical, whilst differences were detected in eight amino acids. The sequences had 94.8% homology.

### 2.2. Core Unique Peptides of Cathelicidin-1

The CUPs identified in cathelicidin-1 of sheep or cattle origin, as well as their exact length and absolute position, are presented in Table S3.

In cathelicidin-1 of sheep origin, 59 CUPs were detected; their median length was 5 (i.e., pentapeptide) (min.–max.: 4–12). In cathelicidin-1 of cattle origin, 73 CUPs were detected; their median length was 6 (i.e., hexapeptide) (min.–max.: 5–13) (*p* < 0.0001 between the length of CUPs in the two proteins) (Table 1). Four identical CUPs were identified in the two proteins (Table 2).

### 2.3. Composite Core Unique Peptides of Cathelicidin-1

**Figure 2:**
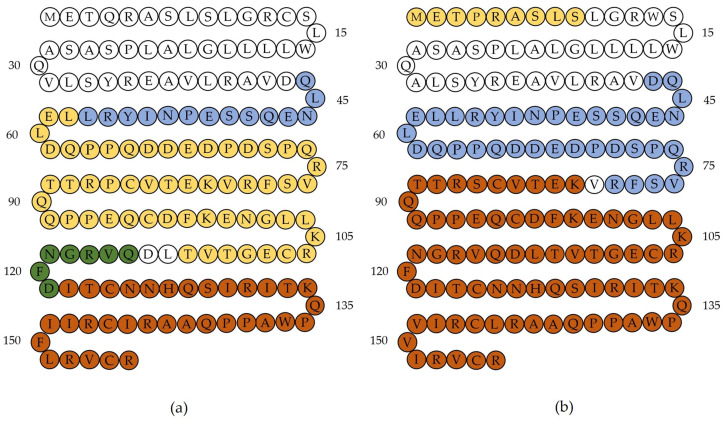
Presentation of composite core unique peptides on the protein sequence of cathelicidin-1 of sheep (**a**) or cattle (**b**) origin: amino acids included in each composite core unique peptide are coloured similarly.

**Figure 3:**
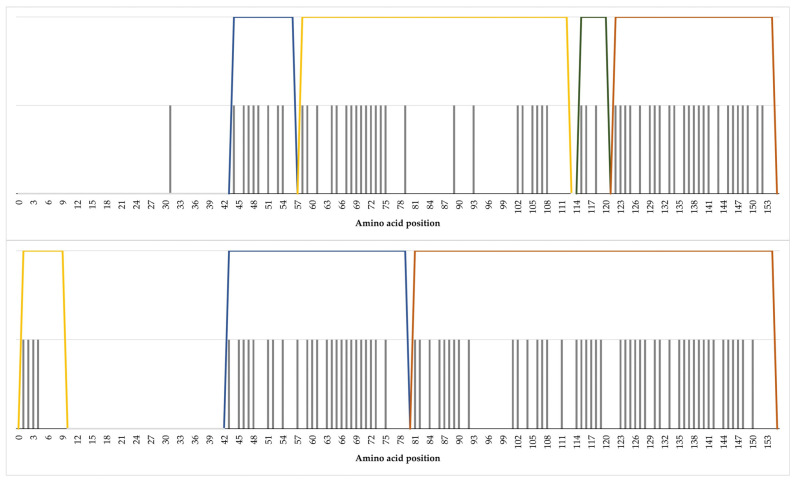
Diagram of composite core unique peptides (overlaying coloured trapezia) with details of the start point of each core unique peptide contained on the protein sequence of cathelicidin-1 of sheep (**upper graph**) or cattle (**lower graph**) origin.

Cathelicidin-1 of sheep origin included four composite core unique peptides (CCUPs), located in positions 44–57, 58–112, 115–121 and 122–155 of the protein sequence. Cathelicidin-1 of cattle origin included three CCUPs in positions 1–9, 43–79 and 81–154 of the protein sequence. Details are presented in Table 3 and Figure 2 and Figure 3.

### 2.4. Unique Peptides of Cathelicidin-1 in Tryptic Digest Peptides

The details of tryptic digest peptides of cathelicidin-1 of sheep or cattle origin are presented in Table S4. Among these, there were six and eight, respectively, unique peptides (Table S4) (*p* = 0.59 for comparison between the two proteins); the six unique peptides found in cathelicidin-1 of sheep origin, were also found in cathelicidin-1 of cattle origin (Table 4), whilst peptide sequences METPR and ETVCSR were found only in cathelicidin-1 of cattle origin (Table S4). Median numbers of CUPs in these were 5.5 (1–9) and 5.5 (3–11), respectively, for cathelicidin-1 of sheep and cattle origin (*p* = 0.75).

### 2.5. Position of Core Unique Peptides of Cathelicidin-1 within its Three-Dimensional Structure

The motifs of the secondary structure of cathelicidin-1 of sheep or cattle origin in all the absolute positions, where CUPs were identified, are detailed in Table S5 and Figure S6.

In cathelicidin-1 of sheep origin, 37 CUPs were found located on the core of the protein (median length: 5) and 22 CUPs were found located at the ends of the two termini of the structure (median length: 4.5) (*p* = 0.22). Among the former, 29 were located on amino acids in regions of the protein with ‘very high’ or ‘confident’ estimates. Among these, 6 CUPs were located entirely on loop or *α*-helix motifs of the secondary structure of the protein (median length: 4.5).

In cathelicidin-1 of cattle origin, 46 CUPs were found located on the core of the protein (median length: 6) and 26 CUPs were found located at the ends of the two termini of the structure (median length: 5, *p* = 0.042). Among the former, 34 were located on amino acids in regions of the protein with ‘very high’ or ‘confident’ estimates and among these, 4 CUPs were located entirely on loop or *α*-helix motifs of the secondary structure of the protein (median length: 5.5).

**Figure 4:**
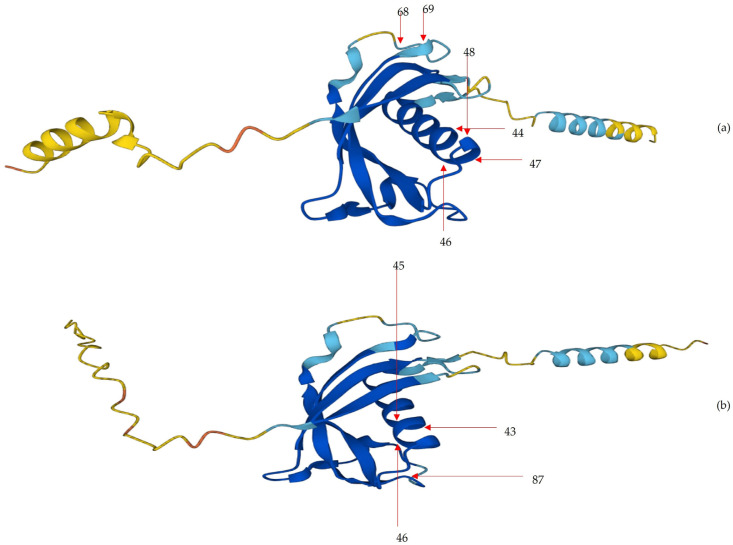
Predicted three-dimensional structure (ribbon model) of cathelicidin-1 of sheep (**a**) or cattle (**b**) origin, indicating also the absolute positions of core unique peptides proposed as potential antigenic targets on the protein structure. Colour code of the protein structure: dark blue ‘very high’ (pLDDT > 90) estimate of confidence, light blue ‘confident’ (90 ≥ pLDDT > 70) estimate of confidence, yellow ‘low’ (70 ≥ pLDDT > 50) estimate of confidence, and orange ‘very low’ (pLDDT ≤ 50) estimate of confidence of the respective structure; red arrows indicate the position of the sequence of Core Unique Peptides. Solid lines indicate that the respective positions of core unique peptides are located on the appearing region of the 3D structure of the protein; numbers indicate the absolute position, i.e., the amino acid position in the protein sequence. Predicted aligned error plots (PAE plots) for the two structures are shown in Figure S5 (model constructed obtained from Uniprot [16,17]; plots in Figure S5 obtained from AlphaFold Protein Structure Database [18,19]).

**Figure 5:**
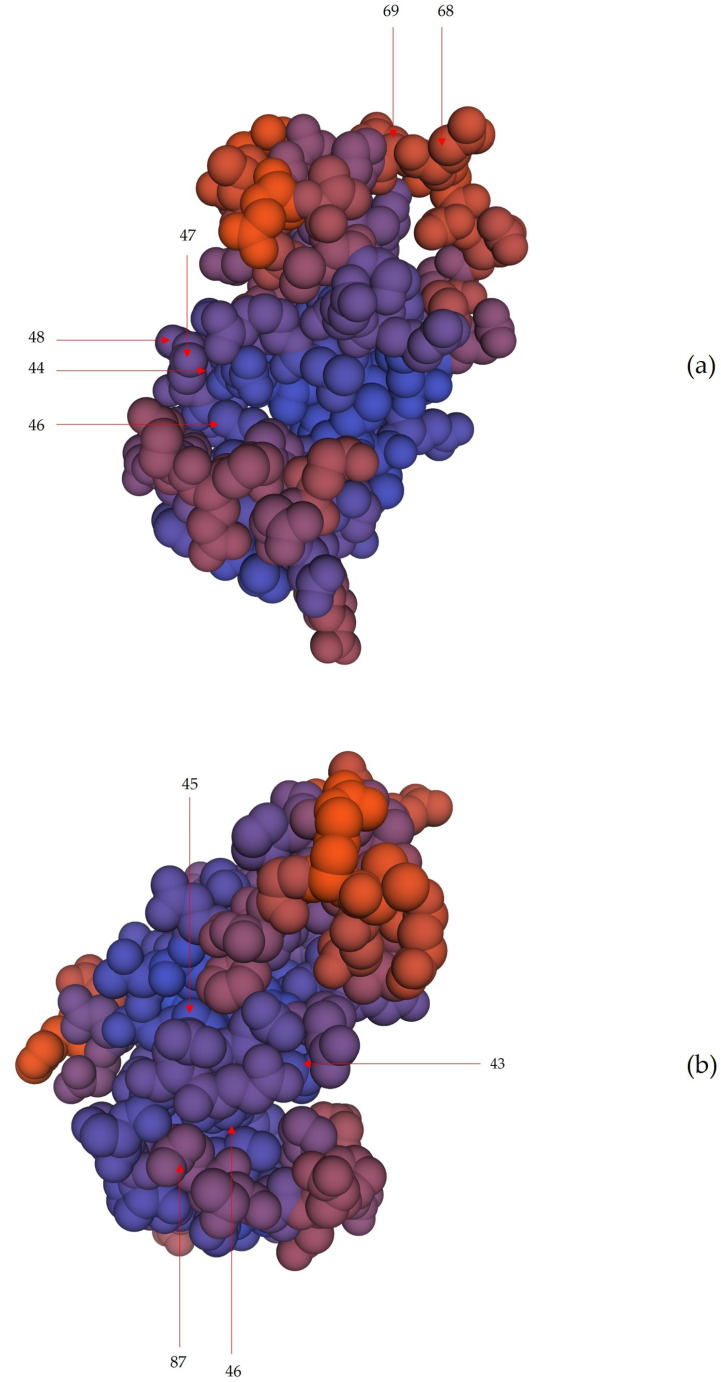
Predicted three-dimensional structure (space-filling model) of cathelicidin-1 of sheep (**a**) or cattle (**b**) origin, also indicating the absolute positions of core unique peptides proposed as potential antigenic targets on the protein structure. Colour code of the protein structure: confidence increases from red- to blue-coloured regions of the protein structure; red arrows indicate the position of the sequence of core unique peptides. Solid lines indicate that the respective positions of core unique peptides are located on the appearing region of the 3D structure of the protein; numbers indicate the absolute position, i.e., the amino acid position in the protein sequence. Model constructed obtained from Swiss-Model [20,21].

The 10 CUPs (6 in cathelicidin-1 of sheep origin and 4 in cathelicidin-1 of cattle origin) located entirely on loop or *α*-helix motifs were found on the outer site of the tertiary structure of the polypeptide backbone of the protein (Figure 4 and Figure 5, Figure S3 and Figure S4). The details of the CUPs proposed as potential antigenic targets for cathelicidin-1 of sheep or cattle origin are in Table 5.

## 3. Discussion

### 3.1. Preamble-Cathelicidins in the Diagnosis of Mastitis in Sheep

Early diagnosis of mastitis is paramount for the successful treatment of the infection. A recent study reported that early instigation of the treatment resulted in earlier complete cure compared to initiating it 24 h later [22]. Effective treatment is important for the welfare of affected animals, as well as for minimizing the risk of development of antibiotic resistance among causal bacteria [23,24].

An array of methods has been proposed for the diagnosis of subclinical mastitis in ewes. These include bacteriological examination of milk samples, cytological study of milk films, mammary imaging, and the detection and identification of biomarkers [25]. Whilst the combination of bacteriological and cytological examination is used as the golden standard for the diagnosis of mastitis [25,26], in clinical practice its application may require some time to perform culturing of the milk samples.

Cathelicidins are released from neutrophils as part of the mammary defence process after bacterial invasion [27]. Moreover, in the mammary gland specifically, the protein was also found to be released by mammary epithelial cells upon the exposure of these cells to the invading pathogens [4,28]. In this context, previous studies have indicated that cathelicidins were present in the milk of animals with mastitis [5], even in the absence of clinical signs [29]. Katsafadou et al. [10] reported that the protein was detected in the milk of affected ewes prior to the increase in somatic cell counts and, moreover, its detection was considered to be sufficient to lead to the diagnosis of subclinical mastitis, as its overall accuracy was found to be >90.0% during the initial 24 h post-infection.

The release of the protein by mammary epithelial cells resulted in its detection in milk as early as 3 h post-infection. This occurred before the influx of neutrophils into the mammary gland. Consequently, there was no need for the quantification of the protein in milk and establishment of a threshold, as a ‘positive’/‘negative’ assessment sufficed [10]. Specifically, in a detailed proteomics analysis of ovine mastitis, which included animal-based experimentations, cathelicidin-1 was only detected in mammary secretion samples collected from inoculated mammary glands within a few hours post-challenge; the protein was not detected in samples obtained from mammary glands of ewes before the challenge, nor in samples from contralateral uninfected mammary glands of the same animals [30].

A further advantage of cathelicidin-1, compared with other inflammation markers, is its lack of detection in the milk of healthy mammary glands [10,30], which lends further support to its use as a biomarker for the diagnosis of mastitis in sheep.

### 3.2. Potential Usefulness of Unique Peptides

The context of unique peptides has been developed by Zhao and Lin [12] with the aim of improving protein identification in mass spectrometry. Indeed, accurate identification of proteins during spectrometry can be challenging [31]. Therefore, the establishment of the unique characteristics of a protein is important for the application of targeted proteomics workflows and peptide/protein-biomarker investigation [13]. These authors further developed the idea of core unique peptides (CUPs), which represent the shortest peptides with a unique sequence [13]. Additionally, the application of unique peptides (and CUPs) in protein identification minimizes the uncertainty of deducing proteins from peptide fragments.

The benefits of using unique peptides in the identification of proteins were first applied in clinical research with the establishment of a biomarker for the early diagnosis of stroke [32]. Subsequent relevant studies have included the development of biomarkers for bacterial respiratory infections [33] and COVID-19 [14,34]. In all those studies, the uniqueness of peptides has facilitated biomarker identification, which, in turn, has led to the successful accomplishment of the performed task. These may refer to the study of pathogenetic pathways, pathogen identification, disease diagnosis, etc. With specific reference to disease diagnosis, the study may involve the identification of peptides in MS-based targeted proteomics [32] or of epitopes for the development of antibodies to accomplish diagnostic objectives.

Additionally to the CUPs, the context of composite core unique peptides (CCUPs) has been devised and reported as they may act as better antigenic targets, given that they consist of more positions. In 2011, i.e., before the spread of the context of unique peptides, Smolenski et al. [29] presented a ‘pan-cathelicidin’ peptide for use in mastitis diagnosis; retrospectively, this could be considered as an early CCUP. Moreover, it is noted that each CUP could produce further unique peptides after adding amino acids thereon.

### 3.3. Application of the Methodology of Unique Peptides for Detection of Cathelicidin-1

The findings of the study have identified a variety of peptides that can be used to detect the presence of cathelicidin-1 in the milk of ewes and consequently to support the diagnosis of mastitis.

The feasibility of using peptides, rather than proteins, for use in the diagnosis of mastitis (through the indirect detection of cathelicidin-1) was shown initially by Smolenski et al. [29]. Thereafter, similar findings were reported by Addis et al. [8,9], who also reported the detection of a pan-cathelicidin peptide sequence.

To enhance the detection of cathelicidin-1, a novel approach was employed in the present study. This involved the use of an advanced bioinformatics tool to detect CUPs [14,15].

In the past, Smolenski et al. [29] reported the development of an (anti-)pan-cathelicidin peptide (CNEQSSEPNIYRLLELDQ), which aligned with a conserved bovine cathelicidin-1 sequence (NEQSSEPNIYRLLELDQ) and aimed to detect the presence of cathelicidin-1 in the milk of cows. By using this sequence, the authors were able to detect cathelicidin-1 before the onset of clinical signs of mastitis, even when somatic cell counts in milk were low [29]. In the present study, the above amino acid sequence was identified at positions 46 to 62 in cathelicidin-1 of sheep and cattle origin (Figure 1) and then, in the detailed analysis, it was found to include eight CUPs for cathelicidin-1 of sheep origin and seven CUPs for cathelicidin-1 of cattle origin.

Thereafter, Addis et al. [8] developed an ELISA in which the pan-cathelicidin peptide sequence was used. Although those authors did not provide the exact sequence of the peptide(s) used, they indicated that this referred to a region of the protein, which aligned 100% with cathelicidin-1, 72% with cathelicidin-2 and 70% with cathelicidin-3 of sheep origin. They also indicated that those peptide(s) aligned 99% with cathelicidin-1 of cattle origin and 68% to 80% with other cathelicidins of cattle origin [9].

It should be noted that, in general, peptides considered for potential use as antigenic targets included ‘conserved’ amino acid regions and would apply to more than one cathelicidin with chemical affinity [29,35,36]. In the present study, further to the above, a different methodological approach was introduced: unique sequences were found at the polypeptide backbone, by means of which one could detect specific protein(s) or groups of proteins in specific species or independently of the species. Then, the exact locations of the unique sequences were studied in relation to the tertiary structure of the protein in order to evaluate their potential use as antigenic targets according to the actual 3D crystal structure that the protein forms, especially given that the function of a protein is dependent on its three-dimensional structure. For this, predicted protein models from AlphaFold were used. AlphaFold was the first computational method to apply machine learning, can predict the 3D structure of a protein from sequence data and can regularly predict protein structures with atomic accuracy, even in cases in which no similar structure is known [37].

During the evaluation of CUPs as possible antigenic targets, only ones detected on the core of the protein according to its 3D structure were selected as these regions indicate more structured and secured regions [38]. Moreover, only sequences on *α*-helices or loops on the protein structure were considered. Various antibodies have been shown to bind onto short *α*-helical peptides present in antigenic proteins, and some of these bind exclusively to a single *α*-helix, with no (or little) interaction with other parts of a protein [39,40,41]. Loops interconnect secondary structural motifs on the surface of a protein, harbour active site residues and are potentially involved in modifying directions of polypeptide chains [42,43,44]. In contrast, *β*-sheets, which are common motifs in protein secondary structure, are involved mostly in steadying the protein structure, fatty-acid binding (required for lipid metabolism), formation of fibrils and protein aggregates, etc. That is, they perform functions that are not related to antigen recognition [45,46,47]; further, Berg et al. [47] and di Vona et al. [48] have indicated that loops may act as potential epitopes.

Further, among *α*-helices and loops, only regions with confident model prediction were considered as potential antigenic targets. A knowledge of the shape and structure of a protein and the recognition of a protein fold with confidence will provide useful cues regarding the function of that molecule. Given the fact that this model is only a predicted one, we chose to work with the most confident sites on the molecule.

Finally, six CUPs identified in cathelicidin-1 of sheep origin and four CUPs identified in cathelicidin-1 of cattle origin have been considered to be more suitable as potential antigenic targets. Among these, three unique peptides were found in both cathelicidin-1 of sheep and cattle origin and can be used in the identification of cathelicidin-1 independently of species of origin. Consequently, CUPs can be used to detect specifically cathelicidin-1 of sheep or cattle origin.

These antigenic targets can be used in the design of specific antibodies for application in techniques, e.g., Western Blot or ELISA, for laboratory use or point-of-care testing for field work. In field work, the early diagnosis of mastitis can be employed to test animals suspected of mastitis, which will thus support the early instigation of treatment and in turn help to cure the infection early [22,49].

For custom antibody production, one strategy uses protein as immunogen. Another approach involves generating short peptides from the native sequence of the target protein for immunization. Such synthetically produced peptide antigens recognize only linear epitopes [50,51,52]. In this respect, it should be noted that the six CUPs proposed as potential antigenic targets (Table 5) in this study fulfil this characteristic.

Moreover, for the development of antibodies against peptide sequences with the aim of detecting native proteins, it is necessary to take into consideration the sequence length, the hydrophilicity/hydrophobicity of peptides, the surface orientation of the protein and the flexibility and the side chains of the residues; these characteristics are important for antibody–antigen interactions. For example, bridging direct hydrogen bonds across the interaction interface contributes to the binding affinity and specificity [53]; further, residues with short hydrophilic side chains (serine, aspartic acid and asparagine) can be enriched alongside the aromatic side chains in the paratopes [54,55,56].

### 3.4. Detection of Unique Peptides in Tryptic Digest Peptides

Mass spectrometry (MS) is a commonly used, high-throughput tool for studying proteins. The procedure of MS-based protein identification employed in bottom-up proteomics involves digesting proteins into peptides using enzymes like trypsin, a serine protease. Trypsin cleaves proteins into peptides with an average size of 700 to 1500 daltons, i.e., within the ideal range for MS [57]. It is highly specific, cutting at the carboxyl side of arginine and lysine residues. The C-terminal arginine and lysine peptides are charged, making them detectable by MS. Trypsin is highly active and tolerant of many additives, with the stringent specificity of trypsin activity being crucial to protein identification; thus, its use is the gold standard for protein digestion by peptides for proteomics. Tryptic digest peptides are separated, fragmented, ionised and captured by mass spectrometers.

The resulting complex mixture of peptides can be identified by tandem mass spectrometry (MS/MS). Proteins are finally identified from the peaks of the captured mass spectra from the tryptic digest peptides using computational methods, where each peak theoretically represents a peptide fragment ion. The dentification of peptides and subsequently of proteins is completed by matching the peptide fragment ion spectra to theoretical spectra generated from protein databases.

However, accurate identification of proteins from tandem mass spectra is challenging, because the main identification approaches include de novo sequencing and database searching [31]. The determination of unique characteristics for a protein is interesting within the application of the new concepts of targeted proteomics workflows [13]. The application is able to search for unique protein fragments derived computationally from enzymatic digestion driven by certain enzymes. Through this application, researchers are able to find unique tags, which are markedly important for protein identification and biomarker discovery. Hence, the concept of unique peptides can be integrated into existing protein identification tools, providing greater accuracy to targeted proteomics. In this way, they are able to further develop the concept of unique peptides into further increasing the confidence of the identified proteins in the MS spectra.

The present findings offer novel mass tags, the use of which would facilitate the detection of cathelicidin-1 during MS-based diagnostics, by offering six unique sequences that will be able to detect cathelicidin-1 of sheep or cattle origin. In this way, this work improves the method of identifying the protein. Moreover, it is noted that tryptic digest peptides with increased number of CUPs would be more stable and resistant to possible mutations than other alternatives, thus being improved choices for mass tags.

## 4. Materials and Methods

### 4.1. Reference Protein Data

Proteomes and proteins of sheep, goats and cattle available were obtained from Uniprot [UniProtKB/SwissProt (release 2022_12)] (UniProt consortium members: European Bioinformatics Institute (EMBL-EBI), Cambridge, United Kingdom; SIB Swiss Institute of Bioinformatics, Geneva, Switzerland; Protein Information Resource, Washington, DC, USA). For the sequence analysis of cathelicidin-1, data from Uniprot were taken into account. Specifically, we only used reviewed cathelicidin-1 of sheep or cattle origin. Νo reviewed cathelicidin-1 of goat origin was available.

A search was only carried out against reviewed proteins. It was considered that, as unreviewed proteome components might contain duplicate registrations and/or unverified sequences and/or protein fragments, unreliable data regarding uniqueness of a protein sequence might be generated; thus, it was decided to exclude unreviewed proteins and proteomes from the search.

### 4.2. Detection of Unique Peptides and Core Unique Peptides of Cathelicidin-1

The presence of unique peptides [12] and of core unique peptides (CUPs) [13,14] was studied in cathelicidin-1 of sheep or cattle origin. The potential uniqueness of each peptide of cathelicidin-1 was investigated against that of all proteins reported to have been detected in sheep, goats or cattle (Section 4.1.), in this way creating a set of CUPs.

The analysis was performed by using a bioinformatics tool built on big data algorithm, as previously detailed [14,15]. In brief, the algorithm receives as input the minimum and maximum peptide length that can be considered as CUPs. Based on those, it creates a rolling window that traverses the sequence of each protein within a given proteome. That way, the algorithm generates a vast amount of data that need to be searched. For a protein of length L with a window of size W, a set with C (= L − W + 1) peptides would be generated. This rolling window delineates a peptide, that may potentially be a core unique peptide. The algorithm must first ensure that the peptide under examination does not contain a previously found CUP from a previous pass of a smaller-sized window. For a window of size N (thus a peptide of the same size), the algorithm checks whether it contains any of the CUPs already identified for that protein for windows sized <N-1. If the peptide under examination would not contain any CUPs, then it is searched against all the other proteins. The process terminates as soon as the peptide is found within any other protein or when all the proteins within the proteome have been examined. The peptide should be considered as a CUP if no other protein contains it.

### 4.3. Detection of Composite Core Unique Peptides of Cathelicidin-1

Peptides that were constructed based on a sequence (continuity or overlapping) of two or more CUPs were considered as composite core unique peptides. The algorithm receives as its input the maximum number of amino acids between two CUPs needed in order for their concatenation to be considered as a composite core unique peptide (CCUP). By definition, this is 0 (zero), which implies that CUPs may overlap or occupy adjacent locations (i.e., distance between them is ≤0 amino acids).

Hence, at least two CUPs would be contained within a CCUP. In the end, a CCUP includes the sequence from the first amino acid of the initial CUP (within that CCUP) to the last amino acid of the final CUP (within that CCUP). This process would avail fewer CCUPs than CUPs within a proteome under evaluation.

### 4.4. Detection of Unique Peptides of Cathelicidin-1 from Tryptic Digest Peptides

The procedure of MS-based protein identification involves digesting proteins into peptides using enzymes such as trypsin. Tryptic digest peptides are separated, fragmented, ionised and captured using mass spectrometers. Proteins are identified from the peaks of the captured mass spectra, using computational methods, and each peak theoretically represents a peptide fragment ion. Trypsin, a serine protease, has become the gold standard for protein digestion to peptides for shotgun proteomics. It is highly specific, cleaving peptide bonds at the carboxyl side of arginine and lysine residues, except for arginine–proline and lysine–proline bonds, which are normally resistant to proteolysis. A stringent specificity of trypsin activity is crucial for protein identification. In order to obtain the peptides resulting from the cleavage of trypsin on cathelicidin-1 in the present study, an algorithm was implemented that simulates the aforementioned rules. It receives a protein sequence as its input and splits that into subsequences wherever it detects an arginine (R) amino acid or a lysine (K) amino acid that is not followed by a proline amino acid (P).

Finally, the tryptic digest peptides, thus obtained, were analyzed as described above (Section 4.2.) in order to identify CUPs in each of their sequences.

### 4.5. Position of Core Unique Peptides of Cathelicidin-1 within its Three-Dimensional Structure

Initially, three-dimensional (3D) cathelicidin-1 structures were retrieved from the AlphaFold database [version 2022-11-01] (European Bioinformatics Institute (EMBL-EBI), Cambridge, United Kingdom). These were created by using the AlphaFold Monomer v2.0 pipeline via the incorporation of novel neural network architectures and training procedures based on the evolutionary, physical and geometric constraints of protein structures [37,58]. The ID AF-P54230-F1 (for cathelicidin-1 of sheep origin) and ID AF-P22226-F1 (for cathelicidin-1 of cattle origin) were used. The database provides differing estimates of confidence (scale: 0–100) for the various regions within the 3D structure for each of the two predicted models, as follows: ‘very high’ (pLDDT > 90), ‘confident’ (90 ≥ pLDDT > 70), ‘low’ (70 ≥ pLDDT > 50) or ‘very low’ (pLDDT ≤ 50) [59]. The 3D structures, obtained as above, were edited by using the ‘cartoon tool’ to depict the polypeptide backbone for ribbon modelling [16,17].

Subsequently, 3D crystal structures of cathelicidin-1 were retrieved from the Swiss-Model Repository database [SMTL version 2023-03-23] (Biozentrum, University of Basel, Basel, Switzerland). These were created by using the Swiss-Model technique. ID P54230 (for cathelicidin-1 of sheep origin) and ID P22226 (for cathelicidin-1 of cattle origin) were used [20,21]. The database provides differing estimates of confidence for the various regions within the 3D structure, which are depicted by different colours and confidence increases from red- to purple- to blue-coloured regions of the protein structure; the average confidence of the entirety of the model of the proteins was 0.73 ± 0.08 for both proteins [60,61,62]. The 3D structures, obtained as outlined above, were edited by using the ‘tool spacefill’ to depict the space-filling model.

### 4.6. Data Management

For each CUP identified within each of the proteins assessed, the following were recorded: length, peptide sequence and absolute position within the protein sequence (i.e., the amino acid position in the protein sequence: start point and end point of the peptide’s first and last amino acid, respectively, in the protein).

For each CCUP identified within each of the proteins assessed, the following were recorded: number of CUPs included, length, peptide sequence and absolute position within the protein sequence. For each protein under evaluation, the number of CUPs and CCUPs found was recorded.

The secondary structure of the polypeptide backbone of cathelicidin-1 of sheep or cattle origin was assessed in detail. The motifs of the secondary structure were recorded [47]. CUPs previously identified were annotated on these elements. Only CUPs (a) in the core of the protein (from position 30 to position 126), (b) in *α*-helices or loops among the motifs of the secondary structure and (c) in regions with ‘very high’ or ‘confident’ estimates, were considered and assessed. CUPs thus selected, were then assessed for their location on the outer or the inner site of the tertiary structure of the polypeptide backbone of the protein, as depicted in a space-filling model [47].

## 5. Conclusions

We investigated sequences in cathelicidin-1 with the objective of identifying its unique peptides and core unique peptides in order to reveal potential targets for the accurate detection of biomarkers. We also sought to find unique sequences among tryptic digest peptides in order to improve the early diagnosis of mastitis in sheep. Ultimately, six core unique peptides, specifically QLNEQ, NEQS, EQSSE, QSSEP, EDPD, DPDS, were identified, and we now propose them as potential antigenic targets for cathelicidin-1 of sheep. Moreover, another six unique peptides were detected in tryptic digests and offer novel mass tags, which would facilitate the detection of cathelicidin-1 during MS-based diagnostics. A subsequent study to assess the interaction patterns between the proposed antigenic targets and recombinant antibodies against these, as well as the relevant molecular dynamics, will be the next step in this project.

## Figures and Tables

**Table 1:** Frequency (*n*) of core unique peptides (CUPs) detected in cathelicidin-1 of sheep or cattle origin, classified according to their length.

Length of CUPs ^1^	CUPs in Cathelicidin-1 of Sheep Origin (*n*)	CUPs in Cathelicidin-1 of Cattle Origin (*n*)
4	26	0
5	29	32
6	0	35
7	2	5
8	0	0
9	0	0
10	0	0
11	1	0
12	2	0
13	0	1
Median	5 ^2^	6 ^2^

^1^ number of amino acids in the peptide; ^2^ *p* < 0.0001 between the two proteins.

**Table 2:** Identical core unique peptides detected in cathelicidin-1 of sheep or cattle origin.

Position ^1^	Sequence of Core Unique Peptides	Length ^2^
59–63	ELDQP	5
75–79	RVSFR	5
107–111	CEGTV	5
116–120	VRGNF	5

^1^ absolute position, i.e., the amino acid position in the protein sequence; ^2^ number of amino acids in the peptide.

**Table 3:** Details of composite core unique peptides found in cathelicidin-1 of sheep or cattle origin.

Position ^1^	Cathelicidin-1 of Sheep Origin			Cathelicidin-1 of Cattle Origin		
	Sequence of Composite Core Unique Peptides	Length ^2^	No. of CUPs ^3^	Sequence of Composite Core Unique Peptides	Length	No. of CUPs
1–9				METPRASLS	9	4
43–79				DQLNEQSSEPNIYRLLE LDQPPQDDEDPDSPKR VSFR	37	24
44–57	QLNEQSSEPNIYRL	14	8			
58–112	LELDQPPQDDEDPDSP KRVSFRVKETVCPRTT QQPPEQCDFKENGLLK RCEGTVT	55	23			
81–154				KETVCSRTTQQPPEQC DFKENGLLKRCEGTVT LDQVRGNFDITCNNH QSIRITKQPWAPPQAA RLCRIVVIRVC	74	45
115–121	QVRGNFD	7	3			
122–155	ITCNNHQSIRITKQP WAPPQAARICRII FLRVCR	34	24			

^1^ absolute position, i.e., the amino acid position in the protein sequence; ^2^ number of amino acids in the peptide; ^3^ CUP: core unique peptide.

**Table 4:** Details of unique peptides found in cathelicidin-1 of sheep origin and in cathelicidin-1 of cattle origin after tryptic digest.

Position ^1^	Sequence of Unique Tryptic Digest Peptides	Length ^2^	No. of CUPs ^3^ Included in Sheep Cathelicidin-1	No. of CUPs Included in Cattle Cathelicidin-1
41–56	AVDQLNEQSSEPNIYR	16	7	6
57–74	LLELDQPPQDDEDPDSPK	18	9	11
88–99	TTQQPPEQCDFK	12	1	3
107–117	CEGTVTLDQVR	11	2	3
118–131	GNFDITCNNHQSIR	14	6	7
135–144	QPWAPPQAAR	10	5	5

^1^ absolute position, i.e., the amino acid position in the protein sequence; ^2^ number of amino acids in the peptide. ^3^ CUP: core unique peptide.

**Table 5:** Details of core unique peptides proposed as potential antigenic targets for cathelicidin-1 of sheep or cattle origin.

Position ^1^	Sequence of CUPs ^2^	Length ^3^	Motifs of Protein Secondary Structure
Cathelicidin-1 of Sheep Origin			
44–48	QLNEQ	5	44–48: *α*-helix
46–49	NEQS	4	46–48: *α*-helix, 49: loop
47–51	EQSSE	5	47–48: α-helix, 49–51: loop
48–52	QSSEP	5	48: *α*-helix, 49–52: loop
68–71	EDPD	4	68–71: loop
69–72	DPDS	4	69–72: loop
Cathelicidin-1 of Cattle Origin			
43–48	DQLNEQ	6	43–48: *α*-helix
45–49	LNEQS	5	45–48: *α*-helix, 49: loop
46–51	NEQSSE	6	46–48: *α*-helix, 49–51: loop
87–91	RTTQQ	5	87–91: loop

^1^ absolute position, i.e., the amino acid position in the protein sequence; ^2^ CUP: core unique peptide; ^3^ number of amino acids in the peptide.

## Data Availability

All data related to this research are available within the manuscript or in the Supplementary Material.

## Supplementary Materials

The supporting information can be downloaded at: https://www.mdpi.com/article/10.3390/ijms241210160/s1.
