# Supplementary material for: Unique Peptides of Cathelicidin-1 in the Early Detection of Mastitis—In Silico Analysis
Source: Int J Mol Sci. 2023 Jun 15;24(12):10160. doi: 10.3390/ijms241210160 (PMC10299713; doi:10.3390/ijms241210160)
Supplement: Supplementary file 1 [file ijms-24-10160-s001.zip › ijms-2392253-supplementary.pdf]

Unique Peptides of Cathelicidin-1 in the Early Detection of Mastitis—In Silico Analysis

Maria V. Bourganou, Evangelos Kontopodis, George Th. Tsangaris, Vasileios Pierros, Natalia G.C. Vasileiou, Vasia S. Mavrogianni, George C. Fthenakis and Angeliki I. Katsafadou

Figure S1. Mean spot densities of cathelicidin-1 in 2-DE gels obtained from sequential milk whey samples from inoculated or uninoculated side of the udder [1], subsequently to inoculation of one gland with *Mannheimia haemolytica* or *Staphylococcus chromogenes* [modified from Katsafadou et al. 2019 [2]].

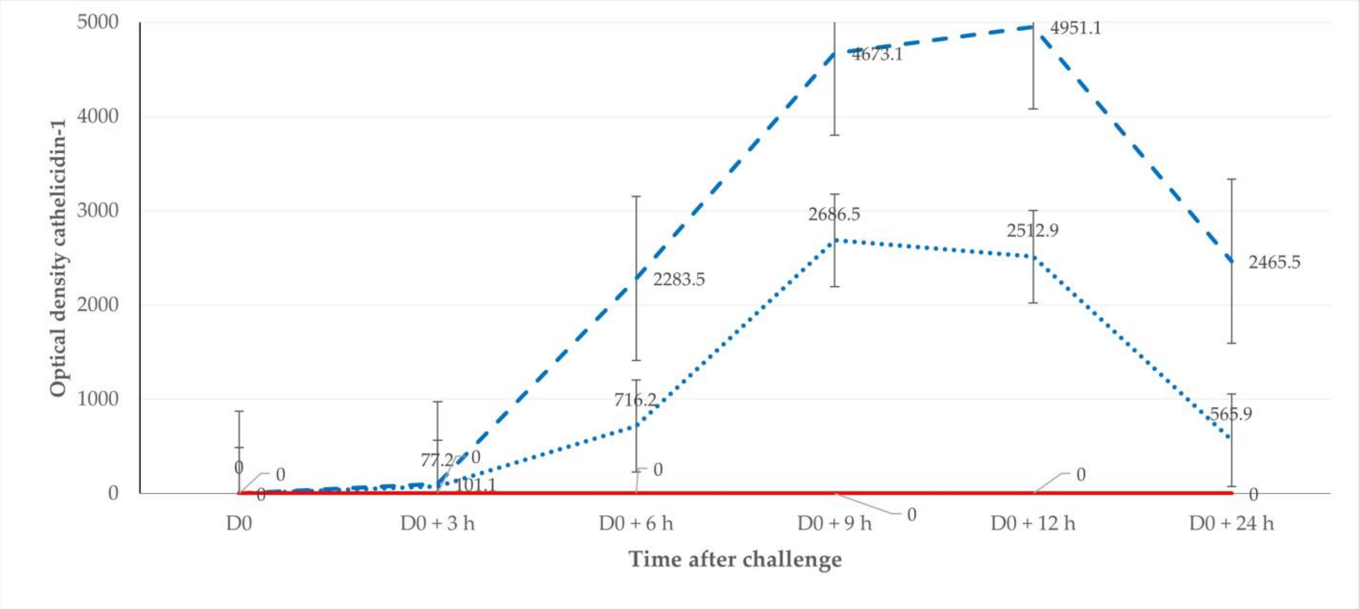

[1] Dashed blue line: glands inoculated with *M. haemolytica*, dotted blue line: glands inoculated with *Staphylococcus chromogenes*), red line: uninoculated glands.

[2] Katsafadou, A.I.; Tsangaris, G.Th.; Vasileiou, N.G.C.; Ioannidi, K.S.; Anagnostopoulos, A.K.; Billinis, C.; Fragkou, I.A.; Papadopoulos, E.; Mavrogianni, V.S.; Michael, C.K.; Addis, M.F.; Fthenakis, G.C. Detection of cathelicidin-1 in the milk as an early indicator of mastitis in ewes. *Pathogens* **2019**, *8*, 270.

Figure S2. Log_{10} of mean spot densities of cathelicidin-1 in 2-DE gels and mean California Mastitis Test scores [1] in sequential milk samples from inoculated side of the udder, subsequently to intramammary infection [2] [modified from Katsafadou et al. 2019 [3]].

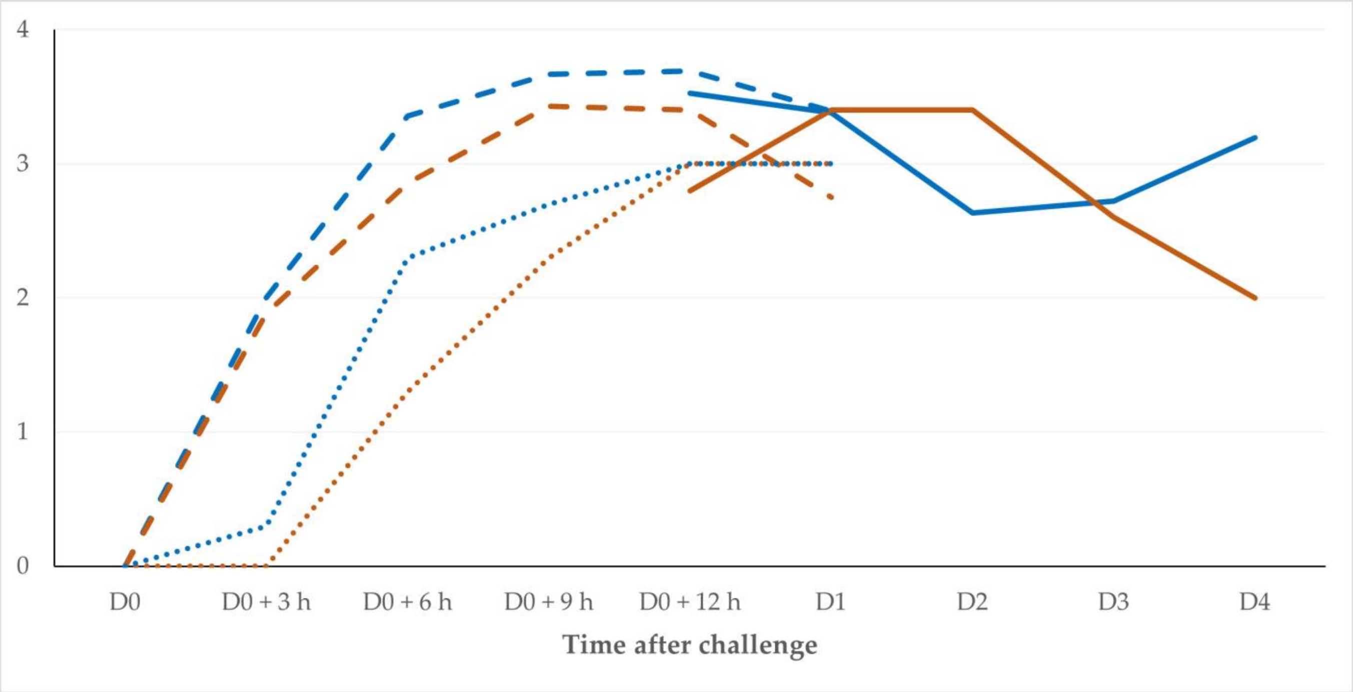

[1] blue lines 2-DE gels, brown lines: California Mastitis Test scores

[2] solid and dashed lines: inoculation with *Mannheimia haemolytica*, dotted lines: inoculation with *Staphylococcus chromogenes*.

[3] Katsafadou, A.I.; Tsangaris, G.Th.; Vasileiou, N.G.C.; Ioannidi, K.S.; Anagnostopoulos, A.K.; Billinis, C.; Fragkou, I.A.; Papadopoulos, E.; Mavrogianni, V.S.; Michael, C.K.; Addis, M.F.; Fthenakis, G.C. Detection of cathelicidin-1 in the milk as an early indicator of mastitis in ewes. *Pathogens* **2019**, *8*, 270.

Table S1. 2 × 2 contingency table indicating number of milk samples from mammary glands with mastitis ('positive' (+) or 'negative' (-)) in relation to presence of cathelicidin-1 therein ('positive' (+) or 'negative' (-)) [modified from Katsafadou et al. 2019 [1]].

(a) Inoculation with *Mannheimia haemolytica*

		Presence of mastitis	
		+	-
Presence of cathelicidin-1	+	20	16
	-	3	51

(b) Inoculation with *Stsaphylococcus chromogenes*

		Presence of mastitis	
		+	-
Presence of cathelicidin-1	+	11	4
	-	0	21

[1] Katsafadou, A.I.; Tsangaris, G.Th.; Vasileiou, N.G.C.; Ioannidi, K.S.; Anagnostopoulos, A.K.; Billinis, C.; Fragkou, I.A.; Papadopoulos, E.; Mavrogianni, V.S.; Michael, C.K.; Addis, M.F.; Fthenakis, G.C. Detection of cathelicidin-1 in the milk as an early indicator of mastitis in ewes. *Pathogens* **2019**, *8*, 270.

Table S2. Characteristics of proteins that were searched for the analysis of cathelicidin-1 against them.

Outcome	Proteins of sheep origin	Proteins of cattle origin	Proteins of goat origin
Proteins (reviewed)	460	6036	120
Proteins with unique peptides	459	6030	118
Core unique peptides in the above proteins	94,011	1,646,928	21,457
Density of core unique peptides	0.66%	0.68%	0.76%
Composite unique peptides	1060	12,459	179
Density of composite unique peptides	0.75%	0.51%	0.63%
Unique coverage	94%	96%	93%

Table S3. Details of core unique peptides found in cathelicidin-1 of sheep or cattle origin.

Start absolute position [1]	Cathelicidin-1 of sheep origin		Cathelicidin-1 of cattle origin	
	Sequence of core unique peptides	Length [2]	Sequence of core unique peptides	Length
1			METPR	5
2			ETPRA	5
3			TPRASL	6
4			PRASLS	6
31	VLSYREAVLRAV	12		
43			DQLNEQ	6
44	QLNEQ	5		
45			LNEQS	5
46	NEQS	4	NEQSSE	6
47	EQSSE	5	EQSSEPN	7
48	QSSEP	5	QSSEPNI	7
49	SSEPN	5		
51	EPNI	4	EPNIY	5
52			PNIYRL	6
53	NIYR	4		
54	IYRL	4	IYRLLE	6
57			LLELDQ	6
58	LELDQ	5		
59	ELDQP	5	ELDQP	5
60			LDQPP	5
61	DQPP	4	DQPPQ	5
63			PPQDD	5
64	PQDD	4	PQDDE	5
65	QDDED	5	QDDEDP	6
66			DDEDPD	6
67	DEDP	4	DEDPDS	6
68	EDPD	4	EDPDSP	6
69	DPDS	4	DPDSPK	6
70	PDSPK	5	PDSPKR	6
71	DSPKR	5	DSPKRV	6
72	SPKRV	5	SPKRVS	6
73	PKRVS	5	PKRVSF	6
74	KRVSF	5		
75	RVSFR	5	RVSFR	5
79	RVKETVCPRTT	11		
81			KETVCS	6
82			ETVCSR	6
84			VCSRT	5
86			SRTTQ	5
87			RTTQQ	5

88			TTQQPP	6
89	TQQPPEQ	7	TQQPPE	6
90			QQPPEQ	6
92			PPEQCDFKENGLL	13
93	PEQCDFKENGLL	12		
101			NGLLKR	6
102	GLLKR	5	GLLKRC	6
103	LLKRC	5		
104			LKRCE	5
105	KRCE	4		
106	RCEG	4	RCEGT	5
107	CEGTV	5	CEGTV	5
108	EGTVT	5	EGTVTLD	7
111			VTLDQVR	7
114			DQVRG	5
115	QVRG	4	QVRGN	5
116	VRGNF	5	VRGNF	5
117			RGNFDI	6
118	GNFD	4	GNFDIT	6
119			NFDITCN	7
122	ITCN	4		
123	TCNN	4	TCNNH	5
124	CNNH	4	CNNHQ	5
125	NNHQ	4	NNHQS	5
126			NHQSI	5
127	HQSI	4	HQSIR	5
128			QSIRIT	6
129	SIRI	4		
130	IRIT	4	IRITK	5
131	RITKQ	5	RITKQP	6
133	TKQP	4	TKQPW	5
134	KQPW	4		
135			QPWAP	5
136	PWAP	4	PWAPPQ	6
137	WAPPQ	5	WAPPQA	6
138	APPQA	5	APPQAA	6
139	PPQAA	5	PPQAAR	6
140	PQAAR	5	PQAARL	6
141	QAARI	5	QAARLC	6
142			AARLCR	6
143	ARICR	5		
144			RLCRI	5
145	ICRI	4	LCRIV	5
146	CRIIF	5	CRIVV	5

147	RIIFL	5	RIVVI	5
148	IIFLR	5	IVVIRV	6
149	IFLRV	5		
150			VIRVC	5
151	LRVC	4		
152	RVCR	4		

[1] absolute position, i.e., the amino acid position in the protein sequence; [2]: number of amino acids in the peptide.

Table S4. Details of unique peptides found in cathelicidin-1 of sheep or cattle origin after tryptic digest.

Cathelicidin-1 of sheep origin				Cathelicidin-1 of cattle origin			
Sequence of tryptic digest peptides	Length [1]	Unique peptides	No of CUPs [2] included	Sequence of tryptic digest peptides	Length	Unique peptides	No of CUPs included
METQR	5	No	0	METPR	5	Yes	1
ASLSLGR	7	No	0	ASLSLGR	7	No	0
CSLWLLLLGLA LPSASAQVLSYR	23	No	0	WSLWLLLLGLA LPSASAQALSYR	23	No	0
EAVLR	5	No	0	EAVLR	5	No	0
AVDQLNE QSSEPNIYR	16	Yes	7	AVDQLNE QSSEPNIYR	16	Yes	6
LLELDQPPQ DDEDPDSPK	18	Yes	9	LLELDQPPQ DDEDPDSPK	18	Yes	11
R	1	No	0	R	1	No	0
VSFR	4	No	0	VSFR	4	No	0
VK	2	No	0	VK	2	No	0
ETVCPR	6	No	0	ETVCSR	6	Yes	1
TTQQPPEQCDFK	12	Yes	1	TTQQPPEQCDFK	12	Yes	3
ENGLLK	6	No	0	ENGLLK	6	No	0
R	1	No	0	R	1	No	0
CEGTVTLDQVR	11	Yes	2	CEGTVTLDQVR	11	Yes	3
GNFDITCNNHQSIR	14	Yes	6	GNFDITCNNHQSIR	14	Yes	7
ITK	3	No	0	ITK	3	No	0
QPWAPPQAAR	10	Yes	5	QPWAPPQAAR	10	Yes	5
ICR	3	No	0	LCR	3	No	0
IIFLR	5	No	0	IVVIR	5	No	0
VCR	3	No	0	VCR	3	No	0

[1] number of amino acids in the peptide; [2]: CUP: core unique peptide.

Table S5. Motifs of secondary structure of cathelicidin-1 of sheep origin in positions, where CUPs were identified.

Start absolute position [1]	Sequence of core unique peptide	Length [2]	Motifs of protein secondary structure
31	VLSYREAVLRAV	12	31-32: β-sheet, 33-42: α-helix
44	QLNEQ	5	44-48: α-helix
46	NEQS	4	46-48: α-helix, 49: loop
47	EQSSE	5	47-48: α-helix, 49-51: loop
48	QSSEP	5	48: α-helix, 49-52: loop
49	SSEPN	5	49-52: loop, 53: β-sheet
51	EPNI	4	51-52: loop, 53-54: β-sheet
53	NIYR	4	53-56: β-sheet
54	IYRL	4	54-57: β-sheet
58	LELDQ	5	58-62: β-sheet
59	ELDQP	5	59-63: β-sheet
61	DQPP	4	61-64: β-sheet
64	PQDD	4	64: β-sheet, 65-67: loop
65	QDDED	5	65-69: loop
67	DEDP	4	67-70: loop
68	EDPD	4	68-71: loop
69	DPDS	4	69-72: loop
70	PDSPK	5	70-72: loop, 73-74: β-sheet
71	DSPKR	5	71-72: loop, 73-75: β-sheet
72	SPKRV	5	72: loop, 73-76: β-sheet
73	PKRVS	5	73-77: β-sheet
74	KRVSF	5	74-78: β-sheet
75	RVSFR	5	75-79: β-sheet
79	RVKETVCPRTT	11	79-86: β-sheet, 87-89: loop
89	TQQPPEQ	7	89-92: loop, 93-95: β-sheet
93	PEQCDFKENGLL	12	93-95: β-sheet, 96: turn, 97-99: β-sheet, 100-103: loop, 104: β-sheet
102	GLLKR	5	102-103: loop, 104-106: β-sheet
103	LLKRC	5	103: loop, 104-107: β-sheet
105	KRCE	4	105-108: β-sheet
106	RCEG	4	106-109: β-sheet
107	CEGTV	5	107-111: β-sheet
108	EGTVT	5	108-111: β-sheet, 112: loop
115	QVRG	4	115-118: loop
116	VRGNF	5	116-119: loop, 120: β-sheet
118	GNFD	4	118-119: loop, 120-121: β-sheet
122	ITCN	4	122-125: β-sheet
123	TCNN	4	123-126: β-sheet
124	CNNH	4	124-126: β-sheet, 127: loop
125	NNHQ	4	125-126: β-sheet , 127-128: loop

127	HQSI	4	127-130: loop
129	SIRI	4	129-132: loop
130	IRIT	4	130-133: loop
131	RITKQ	5	131-135: loop
133	TKQP	4	133-136: loop
134	KQPW	4	134-137: loop
136	PWAP	4	136-137: loop, 138-139: β-sheet
137	WAPPQ	5	137: loop, 138-140: β-sheet, 141: turn
138	APPQA	5	138-140: β-sheet, 141: turn, 142: α-helix
139	PPQAA	5	139-140: β-sheet, 141: turn, 142-143: α-helix
140	PQAAR	5	140: β-sheet, 141: turn, 142-144: α-helix
141	QAARI	5	141: turn, 142-145: α-helix
143	ARICR	5	143-147: α-helix
145	ICRI	4	145-148: α-helix
146	CRIIF	5	146-150: α-helix
147	RIIFL	5	147-151: α-helix
148	IIFLR	5	148-152: α-helix
149	IFLRV	5	149-153: α-helix
151	LRVC	4	151-153: α-helix, 154: turn
152	RVCR	4	152-153: α-helix, 154-155: turn

[1] absolute position, i.e., the amino acid position in the protein sequence.
[2] number of amino acids in the peptide.

Table S6. Motifs of secondary structure of cathelicidin-1 of cattle origin in positions, where CUPs were identified.

Start absolute position [1]	Sequence of core unique peptide	Length [2]	Motifs of protein secondary structure
1	METPR	5	1-4: turn, 5: α-helix
2	ETPRA	5	2-4: turn, 5-6: α-helix
3	TPRASL	6	3-4: turn, 5-8: α-helix
43	DQLNEQ	6	43-48: α-helix
45	LNEQS	5	45-48: α-helix, 49: loop
46	NEQSSE	6	46-48: α-helix, 49-51: loop
47	EQSSEPN	7	47-48: α-helix, 49-52: loop, 53: β-sheet
48	QSSEPNI	7	48: α-helix, 49-52: loop, 53-54: β-sheet
51	EPNIY	5	51-52: loop, 53-55: β-sheet
52	PNIYRL	6	52: loop, 53-57: β-sheet
54	IYRLLE	6	54-59: β-sheet
57	LLELDQ	6	57-62: β-sheet
59	ELDQP	5	59-63: β-sheet
60	LDQPP	5	60-64: β-sheet
61	DQPPQ	5	61-64: β-sheet, 65: loop
63	PPQDD	5	63-64: β-sheet, 65-67: loop
64	PQDDE	5	64: β-sheet, 65-68: loop
65	QDDEDP	6	65-70: loop
66	DDEDPD	6	66-71: loop
67	DEDPDS	6	67-72: loop
68	EDPDSP	6	68-72: loop, 73: β-sheet
69	DPDSPK	6	69-72: loop, 73-74: β-sheet
70	PDSPKR	6	70-72: loop, 73-75: β-sheet
71	DSPKRV	6	71-72: loop, 73-76: β-sheet
72	SPKRVS	6	2: loop, 73-77: β-sheet
73	PKRVSF	6	73-78: β-sheet
75	RVSFR	5	75-79: β-sheet
81	KETVCS	6	81-86: β-sheet
82	ETVCSR	6	82-86: β-sheet, 87: loop
84	VCSRT	5	84-86: β-sheet, 87-88: loop
86	SRTTQ	5	86: β-sheet, 87-90: loop
87	RTTQQ	5	87-91: loop
88	TTQQPP	6	88-92: loop, 93: β-sheet
89	TQQPPE	6	89-92: loop, 93-94: β-sheet
90	QQPPEQ	6	90-92: loop, 93-95: β-sheet
92	PPEQCDFKENGLL	13	92: loop, 93-95: β-sheet, 96: turn, 97-99: β-sheet, 100-103: loop, 104: β-sheet
101	NGLLKR	6	101-103: loop, 104-106: β-sheet
102	GLLKRC	6	102-103: loop, 104-107: β-sheet

104	LKRCE	5	104-108: β-sheet
106	RCEGT	5	106-110: β-sheet
107	CEGTV	5	107-111: β-sheet
108	EGTVTLD	7	108-111: β-sheet, 112-113: loop, 114: β-sheet
111	VTLDQVR	7	111: β-sheet, 112-113: loop, 114: β-sheet, 115-117: loop
114	DQVRG	5	114: β-sheet, 115-118: loop
115	QVRGN	5	115-119: loop
116	VRGNF	5	116-119: loop, 120: β-sheet
117	RGNFDI	6	117-119: loop, 120-122: β-sheet
118	GNFDIT	6	118-119: loop, 120-123: β-sheet
119	NFDITCN	7	119: loop, 120-125: β-sheet
123	TCNNH	5	123-126: β-sheet, 127: turn
124	CNNHQ	5	124-126: β-sheet, 127-128: turn
125	NNHQS	5	125-126: β-sheet, 127-129: turn
126	NHQSI	5	126: β-sheet, 127-130: turn
127	HQSIR	5	127-131: turn
128	QSIRIT	6	128-133: turn
130	IRITK	5	130-134: turn
131	RITKQP	6	131-136: turn
133	TKQPW	5	133-137: turn
135	QPWAP	5	135-139: turn
136	PWAPPQ	6	136-141: turn
137	WAPPQA	6	137-142: turn
138	APPQAA	6	138-143: turn
139	PPQAAR	6	139-144: turn
140	PQAARL	6	140-145: turn
141	QAARLC	6	141-146: turn
142	AARLCR	6	142-147: turn
144	RLCRI	5	144-148: turn
145	LCRIV	5	145-149: turn
146	CRIVV	5	146-150: turn
147	RIVVI	5	147-151: turn
148	IVVIRV	6	148-153: turn
150	VIRVC	5	150-154: turn

[1] absolute position, i.e., the amino acid position in the protein sequence.
[2]: number of amino acids in the peptide.

Figure S3. Predicted three-dimensional structure of part of cathelicidin-1 of sheep, indicating in detail also the positions of core unique peptides (CUPs) proposed as potential antigenic targets on the protein structure (colour code of the protein structure: dark blue 'Very high' (pLDDT > 90) estimate of confidence, light blue 'Confident' (90 ≥ pLDDT > 70) estimate of confidence, yellow 'Low' (70 ≥ pLDDT > 50) estimate of confidence, orange 'Very low' (pLDDT ≤ 50) estimate of confidence of the respective structure; red arrows indicate the position of the sequence of CUPs (solid lines indicate that the respective positions of CUPs are located on the appearing region of the 3-D structure of the protein, dashed lines indicate that the respective positions of CUPs are located beyond the appearing region of the 3-D structure of the protein); numbers indicate the absolute position, i.e., the amino acid position in the protein sequence)) (predicted aligned error plot (PAE plot) for the structure in Figure S5) (model constructed obtained from Uniprot [2023 [1]]).
(a) start positions of CUPs in 44th, 46th and 47th amino acid position in the protein sequence; (b) start positions of CUPs in 68th and 69th amino acid position in the protein sequence.

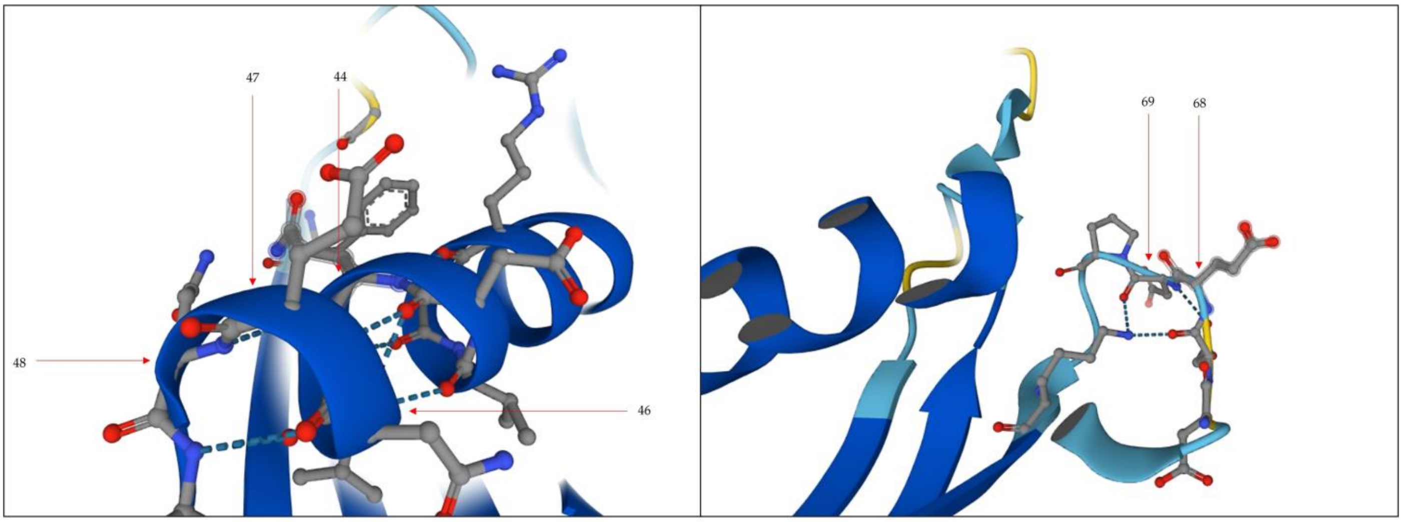

(a) (b)

[1]: UniProt. P54230 CTHL1_SHEEP, 2023, https://www.uniprot.org/uniprotkb/P54230/entry (accessed on 18 April 2023).

Figure S4. Predicted three-dimensional structure of part of cathelicidin-1 of cattle, indicating in detail also the positions of core unique peptides (CUPs) proposed as potential antigenic targets on the protein structure (colour code of the protein structure: dark blue 'Very high' (pLDDT > 90) estimate of confidence, light blue 'Confident' (90 ≥ pLDDT > 70) estimate of confidence, yellow 'Low' (70 ≥ pLDDT > 50) estimate of confidence, orange 'Very low' (pLDDT ≤ 50) estimate of confidence of the respective structure; red arrows indicate the position of the sequence of CUPs (solid lines indicate that the respective positions of CUPs are located on the appearing region of the 3-D structure of the protein, dashed lines indicate that the respective positions of CUPs are located beyond the appearing region of the 3-D structure of the protein); numbers indicate the absolute position, i.e., the amino acid position in the protein sequence)) (predicted aligned error plot (PAE plot) for the structure in Figure S5) (model constructed obtained from Uniprot [2023 [1]]).
(a) start positions of CUPs in 43rd, 45th and 46th amino acid position in the protein sequence; (b) start positions of CUPs in 46th amino acid position in the protein sequence; (c) start positions of CUPs in 87th amino acid position in the protein sequence.

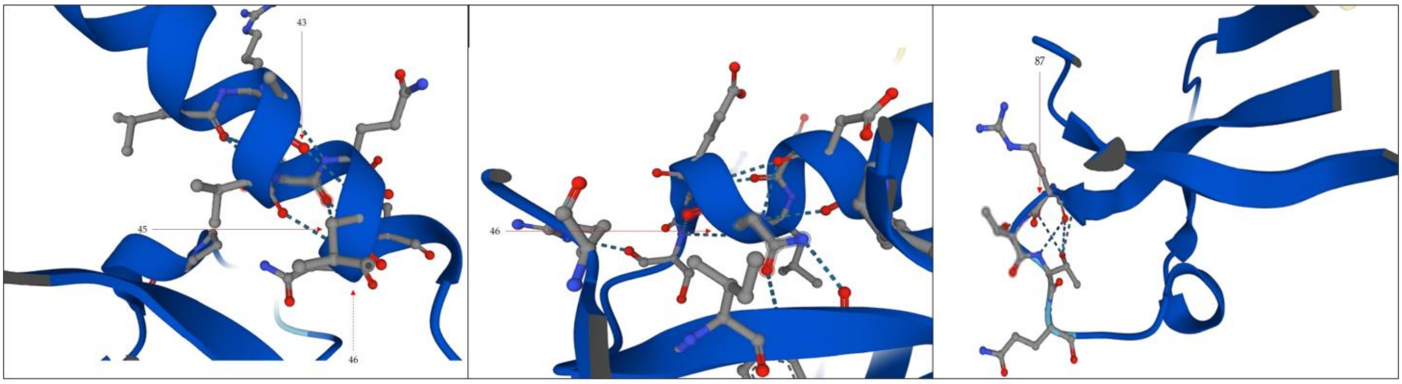

(a) (b) (c)

[1]: UniProt. P22226 CTHL1_BOVIN, 2023, https://www.uniprot.org/uniprotkb/P22226/entry (accessed on 18 April 2023).

Figure S5. Predicted aligned error plot (PAE plot) of the three-dimensional structure (ribbon model) of cathelicidin-1 of sheep (a) or cattle (b) origin (plot obtained from AlphaFold Protein Structure Database [2023 [1,2]]).

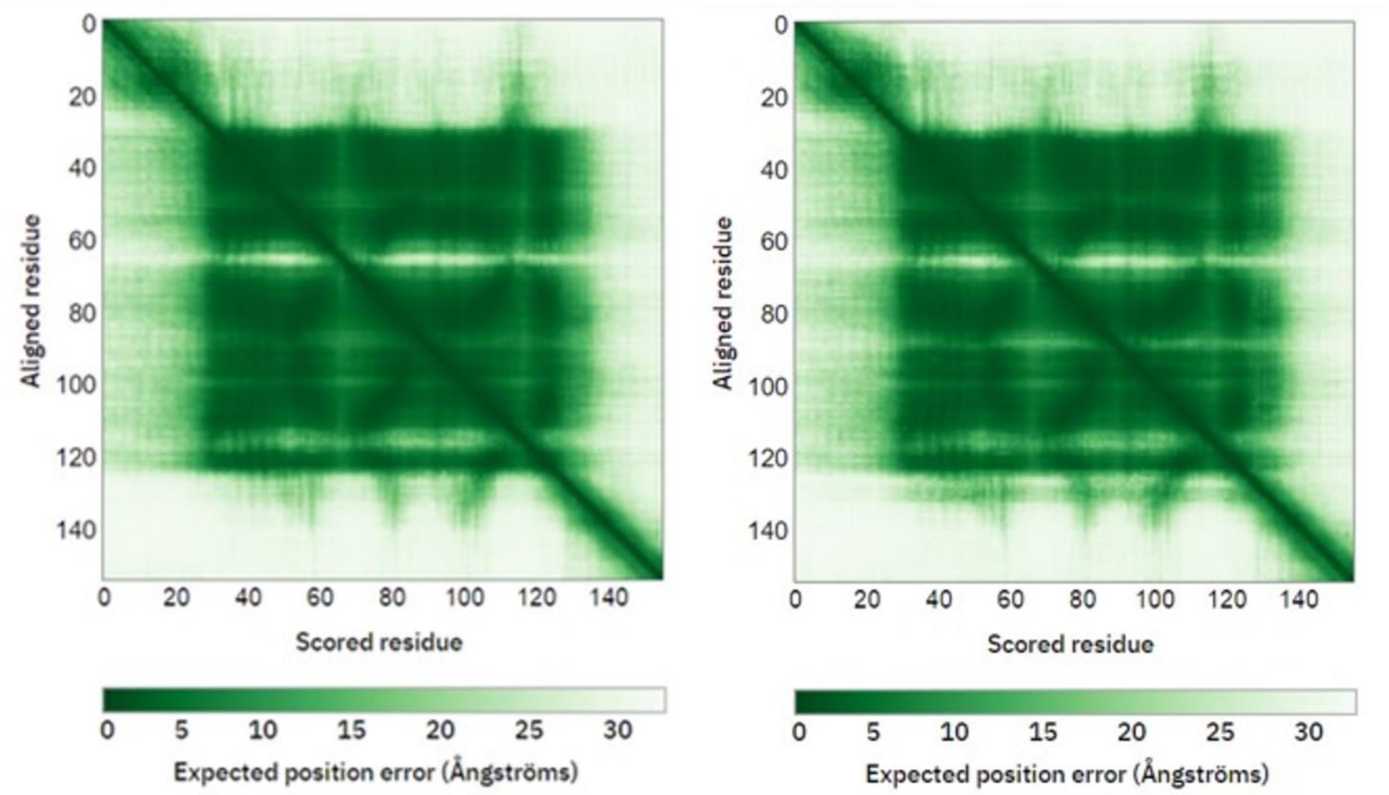

(a) (b)

[1]: AlphaFold Protein Structure Database. Cathelicidin-1 AlphaFold structure prediction, 2023, https://alphafold.ebi.ac.uk/entry/P54230?fbclid=IwAR0bPMzS62Q0j8hB-8Wr9n8f99ww29M8pkf0L_taYWprz8KrzG59GkaDSLU (accessed on 24 May 2023).

[2]: AlphaFold Protein Structure Database. Cathelicidin-1 AlphaFold structure prediction, 2023, https://alphafold.ebi.ac.uk/entry/P22226?fbclid=IwAR31Qa_xJkxfjAI0sCZ9_Phar4DZv12w8WcqIJT3Fte3g4C4iFWfxYNF1sk (accessed on 24 May 2023).
